# Rapid adaptation to high temperatures in *Chironomus riparius*

**DOI:** 10.1002/ece3.4706

**Published:** 2018-12-03

**Authors:** Quentin Foucault, Andreas Wieser, Ann‐Marie Waldvogel, Barbara Feldmeyer, Markus Pfenninger

**Affiliations:** ^1^ Molecular Ecology Group Senckenberg Biodiversity and Climate Research Centre Frankfurt am Main Germany; ^2^ Institute for Organismic and Molecular Evolution Johannes Gutenberg Universität Mainz Germany

**Keywords:** acclimation, Chironomidae, climate, developmental temperature, ectotherm, temperature adaptation

## Abstract

Effects of seasonal or daily temperature variation on fitness and physiology of ectothermic organisms and their ways to cope with such variations have been widely studied. However, the way multivoltines organisms cope with temperature variations from one generation to the next is still not well understood. The aim of this study was to investigate whether the multivoltine midge *Chironomus riparius* Meigen (1803) responds mainly via acclimation as predicted by current theories or whether rapid genetic adaptation is involved. To investigate this issue, a common garden approach has been applied. A mix of larvae from five European populations was raised in the laboratory at three different pre‐exposure temperatures (PET): 14, 20, and 26°C. After three and five generations, respectively, larvae were exposed to three treatment temperatures (TT): 14, 20, and 26°C. Mortality was monitored for the first 48 hr and after emergence. After three generations, significant mortality rate differences depended on an interaction of PET and TT. This finding supports the hypothesis that chironomids respond rapidly to climatic variation via adaptive mechanisms and to a lesser extent via phenotypic plasticity. The result of the experiment indicates that three generations were sufficient to adapt to warm temperature, decreasing the mortality rate, highlighting the potential for chironomids to rapidly respond to seasonally changing conditions.

## 1 INTRODUCTION

Ambient temperature variation is a major factor affecting the fitness of organisms by regulating the speed of metabolic processes, and thus, everything from development to reproduction (Atkinson, 1994). Therefore, local thermal regimes impose strong selection pressures on organisms (Merilä & Hendry, 2014; Waldvogel et al., 2018). Ectothermic organisms are particularly affected, due to their dependence of body temperature from ambient temperature (Clarke & Fraser, 2004; Deutsch et al., 2008). In ectotherms, metabolic rates show a more or less linear temperature response over a large temperature range; however, below and above a species‐specific threshold, physiological performance rapidly drops. In contrast, the fitness of ectotherms displays an exponential increase with temperature. The mortality curve takes on a U shape, skewed toward higher temperatures with a maximal fitness at certain, optimal temperature (*T*_opt_). The edges of this curve are characterized by a steep drop of survival at a certain low and high temperature (*T*_crit_) (Regniere, Powell, Bentz, & Nealis, 2012) that usually coincides with the drop in physiological performance. Therefore, temperature‐related mortality in ectotherms seems mostly driven by physiological limitations (Paaijmans et al., 2013; Pörtner, 2001; Verberk, Calosi, Spicer, Kehl, & Bilton, 2017). In temperate areas, seasonal and daily temperature variation is believed to select for organisms with broad thermal tolerances following Janzen's climate variability hypothesis (Janzen, 1967; Shah, Ghalambor, & Shah, 2017). This can be achieved, for example, by morphological modifications of body size depending on temperature to adjust energy requirements, known as Bergmann's rule (French, Feast, & Partridge, 1998; Gardner, Peters, Kearney, Joseph, & Heinsohn, 2011), via both physiological plasticity and genetic adaptation (Angilletta, Steury, & Sears, 2004; Atkinson & Sibly, 1997). An example for a purely physiological organismic response to short exposure periods of both cold and heat stress is hardening mechanisms involving protein modifications at the cell level (Bowler, 2005; Overgaard, Sørensen, Petersen, Loeschcke, & Holmstrup, 2005). Therefore, effect of temperature variation on plastic life history traits such as growth (Frouz, Ali, & Lobinske, 2002; Hauer & Benke, 1991), reproduction (Péry & Garric, 2006), and metabolic processes (Edwards, 1958; Hirthe, Fisher, Crane, & Callaghan, 2001; Sankarperumal & Pandian, 1991) is well known.

Even though the effects of short‐term temperature variations on physiology and fitness have been well studied, the processes governing responses to short‐term seasonal temperature variations on ectothermic organisms are still not fully understood (Paaijmans et al., 2013). This is in particular true for short‐lived, multivoltine species whose successive generations are exposed to highly different temperature regimes throughout the year, at least in temperate regions with their pronounced seasonality.

To cope with such a broad temperature variation, likely exceeding their optimal temperature range at least in the extremes, multivoltine ectothermic organisms can respond in different ways, such as (a) behavioral changes, allowing organisms to escape or mitigate the environmental pressure (Hutchison & Maness, 1979; Lencioni, 2004); (b) phenotypic plasticity, resulting in changes in gene expression to alleviate abiotic stress (Johnston & Wilson, 2006); and/or (c) by genetic adaptation (Bergland, Behrman, O'Brien, Schmidt, & Petrov, 2014) to maximize their fitness. It is commonly assumed that (a) and (b) act rather on short timescales, while (c) was presumed to proceed over long timescales (Carroll, Hendry, Reznick, & Fox, 2007). However, it becomes increasingly clear that evolutionary adaptation can be very rapid (Messer & Petrov, 2013) and can even occur on seasonal timescales (Bergland et al., 2014).

Because both phenotypic plasticity and rapid adaptation are necessarily associated with changes in the phenotype, distinguishing whether a change in phenotype is genetically based or results only from phenotypic plasticity is difficult. It requires experimental testing over several generations in order to identify which of those mechanisms are involved. To this end, we performed a common garden experiment including different thermal environments with transplantation experiments after three and five generations of exposure to fixed temperature. This method allows us to compare the mortality of the individuals depending on which temperature the parents come from and the temperature experienced by the offspring (Gienapp, Teplitsky, Alho, Mills, & Merilä, 2008; Merilä & Hendry, 2014). This procedure also permits us to identify possible transgenerational effects by comparing the mortality at the temperature the parents were raised (Fox & Mousseau, 1998; Valtonen, Kangassalo, Pölkki, & Rantala, 2012), as well as possible hardening mechanism by recording the mortality 24 hr after hatching (Bowler, 2005; Sinclair & Roberts, 2005). If the response to different temperatures is governed by phenotypic plasticity, we expect the phenotype and the resulting fitness of each generation to depend only on the temperature experienced by the according generation, independent of the temperature experienced by previous generations. Thus, irrespective of generation, individuals should perform similarly well in each temperature (i.e., according to the respective reaction norm for this temperature). If, however, genetic adaption occurred, the phenotypic fitness response to different temperatures will rather depend on the temperature experienced by the previous generation(s), that is, organisms should perform better in temperatures their progenitors were exposed to. Consequently, we expect an interaction between temperatures experienced by previous generations and temperatures experienced by the test individuals. Therefore, this procedure also allows us to distinguish between adaptation and transgenerational effect such as parental effect (Burgess & Marshall, 2014). The aim of this study was to infer how an ectothermic multivoltine invertebrate, *Chironomus riparius* (MEIGEN 1803) (Supporting Information Figure S1), spending most of its life in an aquatic larval stage (Armitage, Cranston, & Pinder, 1995), copes with the short‐term seasonal temperature variations over a few generations—via phenotypic plasticity or rapid adaptation. The species fares very well with experimental culture conditions over several generations (Downe & Caspary, 1973; Nowak, Jost, et al., 2007; OECD, 2004; 2010) and is therefore well suited for evolutionary experiments. Moreover, the adaptive potential of this species to a large gradient of local climate conditions has already been shown (Nemec, Patel, Nowak, & Pfenninger, 2013; Waldvogel et al., 2018). Wild populations of *C. riparius* harbors ample genetic variation and have a large effective population size (~10^6^, (Oppold & Pfenninger, 2017)). In addition, the high number of offspring per breeding pair likely render selection processes on quantitative traits very effective (Pfenninger, 2017). In this study, we hypothesize therefore that *C. riparius* can genetically adapt rapidly within a few generations to different temperature regimes, which would give first experimental evidence for rapid adaptation to seasonal temperature fluctuations.

## 2 MATERIALS AND METHODS

**Figure 1:**
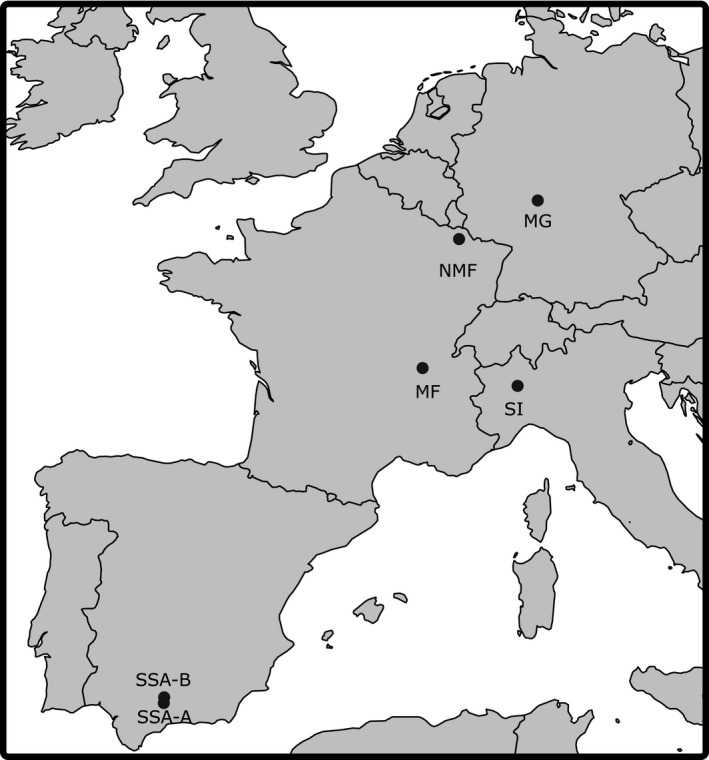
Map of the sampling site of the mixed population

This experiment was performed on an admixed *C. riparius* culture composed of individuals from five different populations originating from Metz (NMF) and Lyon (MF) in France, Hasselroth in Germany (MG), Collobiano in Italy (SI), and Las Vegas in Spain (SS) (Oppold et al., 2016) (Figure 1). These populations were kept under laboratory conditions for a minimum of three generations prior to admixture. We decided to mix individuals from multiple populations in order to compensate for the expected loss of variation due to laboratory selection effect during the experiment (Nowak, Vogt, Diogo, & Schwenk, 2007) and to make sure that alleles involved in adaptation to the complete temperature gradient are present in the gene pool. This setup nevertheless is also raising the problem of possible assortative mating since individuals from different population could prefer individuals from the same population. This possible bias is regarded as negligible after three generations considering the size of the cage forcing reproduction between individuals from different populations. From this admixed base population, which was kept for one generation at 20°C, three subpopulations were created. Two replicates from each subpopulation were raised at three different temperatures: 14, 20, and 26°C with light–dark rhythm of 16:8 hr at 60% humidity, following the OECD guideline 219 (OECD, 2004; Oppold et al., 2016). These temperatures were oriented at the maximum, average, and minimum mean temperature recorded during the meteorological summer (26.9°, 20.9°, and 15.3°C) for the five populations of origin (Waldvogel et al., 2018). Those values were adjusted to obtain constant 6°C differences among experimental treatments. Field measured temperatures have been used to avoid reaching critical temperatures that are not commonly experienced in the wild, avoiding possible bias on the recorded mortality by artificially causing bottlenecks in the population (Supporting Information Table S1).

Larvae were raised in medium constituted of purified water with sea salt (Tropic Marin) adjusted to a conductivity of 520–540 µS/cm and pH 8. The bottom of the glass bowl (20 cm diameter) was covered with washed sand. Populations were raised at these temperatures for three and five generations to simulate the possible seasonal exposure range (i.e., number of generations which could be expected during meteorological summer); this phase will hereafter be referred to as “Pre‐Exposure Temperature” (PET).

### 2.1 Survival (mortality) test

Survival tests were common garden experiments performed in the third and fifth generation, that is, individuals were exposed to PET for three and five generations, respectively. These two different time points were used to investigate possible acclimation or adaptation during the expected high and low number of generations possible during the meteorological summer. Generation time at the respective temperature was determined during the first generation, as time from the hatching of the eggs until the death of the adults. This was necessary, because generations started overlapping with the second generation, making it impossible to infer the exact beginning and end of a generation. The generation times used were inferred from their emergence time calculated in laboratory experiments: 33.6 days at 14°C, 18.1 days at 20°C, and 11.4 days at 26°C (Oppold et al., 2016).

**Figure 2:**
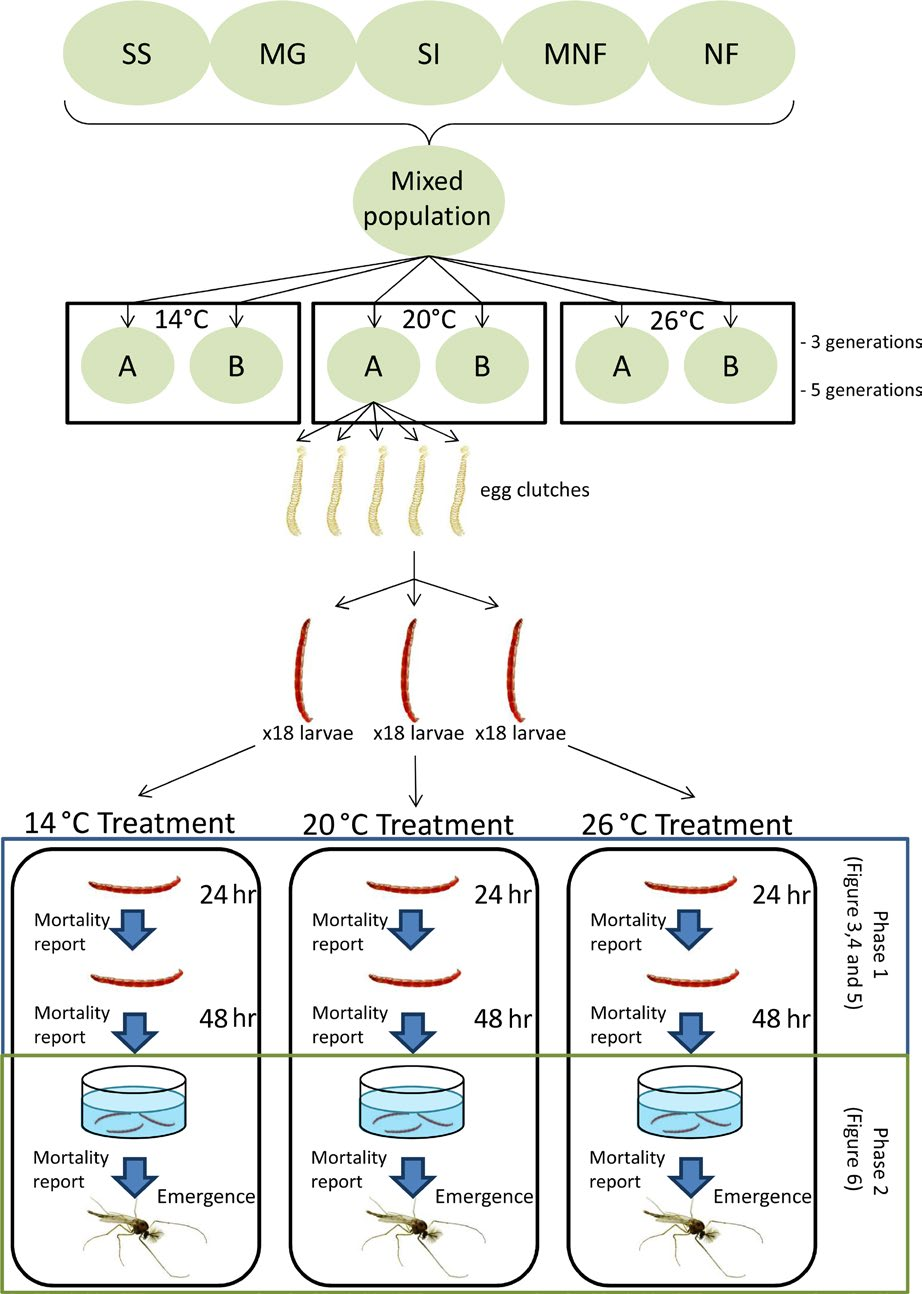
Schema of the experimental design used to record mortality rate depending on temperature using a common garden approach

For each replicate, five egg clutches were put to hatch. Individuals coming from the same egg clutch will be referred to as families. Eighteen larvae from each family of each replicate were raised at three different experimental temperatures called “Treatment Temperature” (TT) for the analysis (18 larvae × 5 families × 3 temperatures × 2 population replicates) (Figure 2). Each larva was individually raised in six‐well plates (Ø3.5 × 2 cm) filled with 6 ml of medium for 48 hr without feeding to avoid altering the medium. Larval mortality rates were measured first after 24 and then 48 hr. After 48 hr, the surviving larvae were pooled by families in glass bowls (Ø20 × 10 cm) with sediment and medium and reared until emergence. The mortality of pupation and emergence was calculated as the number of individuals not emerged per family, for each combination of PET and TT, when adults were removed from bowls. During this stage, larvae were fed daily with dried fish food (0.5 mg/individual of grounded TetraMin^®^ flakes) and the water level adjusted daily with deionized water in order to conserve the physicochemical parameters.

### 2.2 Statistics

Statistical analyses were performed using R (Version 3.2.3) in addition with Rstudio (Version 0.99.903) using lme4 and nlme package for linear mixed‐effects model and the package lsmeans and PMCMR for post hoc tests. The normality of the data set was tested using Q‐Q plot, Kolmogorov–Smirnov tests, and homoscedasticity with Levene's test. A linear mixed model was used to investigate the effect of the PET, TT, and generation as well as their interactions on mortality with the families per PET (clutches) as random factor followed by ANOVA tests.

In case of significant interactions of two or more factors, each instance of the interaction was analyzed separately. In order to investigate significant differences between the TTs, ANOVA followed by Tukey post hoc tests was used for data following the assumptions of normality and homoscedasticity. Kruskal–Wallis tests followed by Dunn post hoc tests were used if one or both of the previous assumptions were rejected.

## 3 RESULTS

### 3.1 Larval mortality

**Figure 3:**
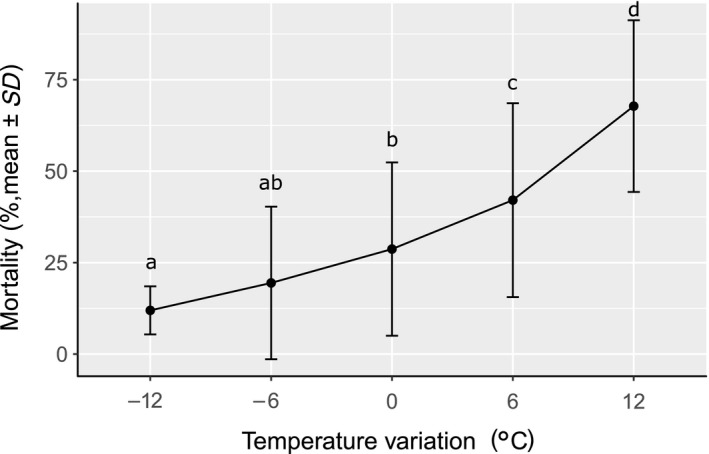
Mean larval mortality after 48 hr depending on the delta temperature (°C) (delta temperature = TT–PET). Letters denote significant mortality differences between TTs (Kruskal–Wallis $\chi_{4,175}^{2}$ = 67.320, *p*‐value = 8.347 × 10^−14^)

Larval mortality was significantly affected by pre‐exposure temperature (PET) and test temperature (TT), with a significant interaction between these two factors. However, larval mortality did not differ significantly between the third and the fifth generations or between replicates. Based on this result, both generations and replicates were grouped for further statistical analyses (Table 1), and data for each generation can be found in the Supporting Information. Mean larval mortality increased with the increase in delta temperature between pre‐exposure and experimental temperature (Δ*T*°= TT–PET) (Kruskal–Wallis $\chi_{4,175}^{2}$ = 57.253, *p*‐value = 1.1 × 10^−11^) (Figure 3).

**Table 1:** Summary of the linear mixed model executed for the larval mortality after 48 hr at TT

Factor	*df*	Sum Sq	Mean Sq	*F* value	Pr(>*F*)
PET	1	1732.8	1732.8	140.198	<2e^−^^16^***
TT	1	273.0	273.0	21.786	6.11e^−^^06^***
Generation	1	24.2	24.2	1.931	0.166
PET*TT	1	248.5	248.5	19.831	1.52e^−^^05^***
Generation*PET	1	28.0	28.0	2.237	0.1370
Generation*TT	1	1.4	1.4	0.112	0.738
Generation*PET*TT	1	0.6	0.6	0.049	0.825
Residuals	172	2155:4	12.5		

With generation, PET, and TT as fixed factor and clutches (replicate per PET) as random factor.

The significance level of the *p*‐value is displayed as the number of stars (***p‐values < 0.001).

**Figure 4:**
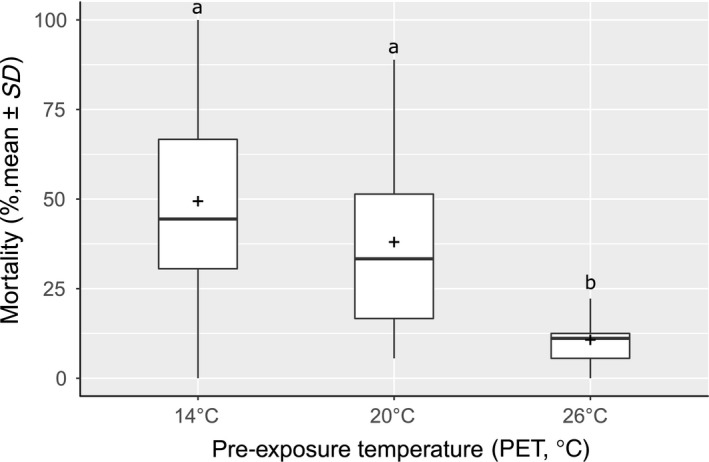
Boxplot of larval mortality after 48 hr depending on the PET (°C), the crosses show the mean mortality for each PET. The letters denote significant differences between mortalities (Kruskal–Wallis $\chi_{2,59}^{2}$ = 79.513, *p*‐value < 2.2 × 10^−16^)

**Figure 5:**
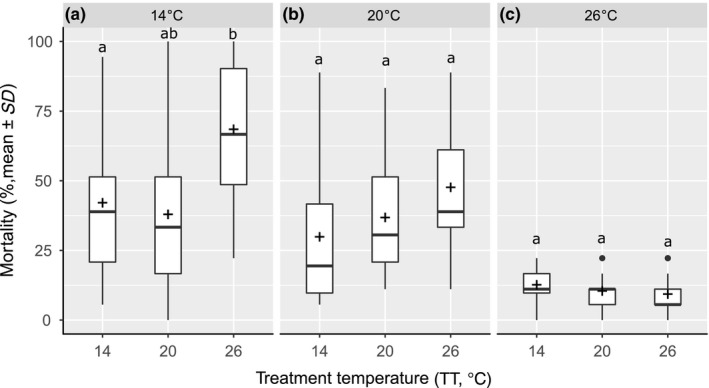
Boxplot displaying larval mortality after 48 hr by TT (°C) for (a) PET 14°C (ANOVA *F*_2,57_ = 15,18, *p*‐value = 5.19 × 10^−6^), (b) PET 20°C (ANOVA *F*_2,19_ = 2.936, *p*‐value = 0.06), and (c) PET 26°C (ANOVA *F*_2,19_ = 1.453, *p*‐value = 0.24). Crosses illustrate mean mortality for each PET. Different letters denote significant differences in mortality between groups

After 48 hr, significant differences were found between larval mortalities depending on the PET (Kruskal–Wallis $\chi_{2,177}^{2}$ = 76.932, *p*‐value <2.2 × 10^−16^).The overall mortality of the larvae was significantly lower for individuals coming from PET_26° _compared to a PET_14°_ (post hoc Dunn's test *p*‐value = <2 × 10^−16^) or PET_20°_ (post hoc Dunn's test *p*‐value = 7.2 × 10^−10^) (Figure 4), independently of TT. By looking at TT depending on the PET, larvae reared at PET_14°_ showed different mortality between TTs (ANOVA *F*_2,57_ = 8.889, *p*‐value = 7.4 × 10^−4^): Mortality was significantly higher at TT_26° _than at both TT_20°_ (post hoc Tukey's test *p*‐value = 3.6 × 10^−5^) and TT_14°_ (post hoc Tukey's test *p*‐value = 54.1 × 10^−5^) (Figure 5a). For the larvae from PET_20°_, none of the TT showed a significant difference for larval mortality compared to the PET (ANOVA *F*_2,57_ = 2.936, *p*‐value = 0.06), but a replicated regression showed a significant linear relation between the TTs (*F*_2,57_ = 3.672845, *p*‐value = 0.0317) (Figure 5b). Finally, for the larvae coming from the PET_26°_, no significant difference was found between the different TTs, even though the means showed a trend for lower mortality at higher temperatures (ANOVA *F*_2,57_ = 1.453, *p*‐value =0.24) (Figure 5c).

**Figure 6:**
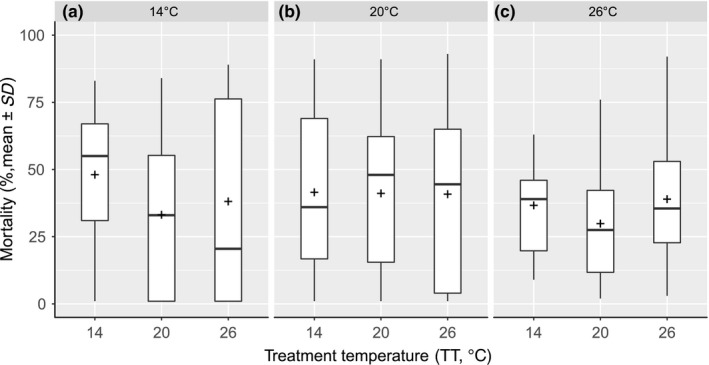
Boxplot displaying the pupae mortality by TT (°C) for (a) PET 14°C (Kruskal–Wallis $\chi_{2,19}^{2}$ = 2.226, *p*‐value = 0.3), (b) PET 20°C (ANOVA *F*_2,57_ = 0.002, *p*‐value = 0.9), and (c) PET 26°C (ANOVA *F*_2,57_ = 1.012, *p*‐value = 0.3). Crosses show the mean mortality rate for each PET

The pupae mortality was not significantly different between the any of the TTs (ANOVA *F*_2,177_ = 1.023, *p*‐value = 0.36) or PETs (PET_14°_:Kruskal–Wallis $\chi_{2,57}^{2}$ = 2.226, *p*‐value = 0.3, PET_20°_:ANOVA *F*_2,57_ = 0.007, *p*‐value = 0.9, PET_26°_: ANOVA *F*_2,57_ = 1.575, *p*‐value =0.4) (Figure 6).

## 4 DISCUSSION

The aim of this study was to investigate the possibility of rapid adaptation of *C. riparius* to seasonal temperature changes. Experimental populations, composed of individuals from five natural populations across a wide distribution range, were reared under three different temperature regimes and mortality rates tested after the third and fifth generations, at the same three temperatures, respectively. The pupation mortality did not display any significant differences between treatments while having an important mortality which is consistent with pupation being the most critical life stage (Oliver, 1971). The insensitivity of pupal mortality to external factors suggests that rather internal factors and processes are responsible for this mortality. For this reason, only larval mortality is relevant here. Pre‐exposure over three generations to the lowest mean summer temperature experienced by either of the five natural populations composing the experimental populations did not have an influence on larval mortality at low and intermediate experimental temperatures (Figure 4). It did, however, increase susceptibility to high temperatures resulting in the highest recorded mortality in our experiments (Figure 5, left panel) which could be explained by the increase in cold‐adapted alleles and therefore a lower adaptation to heat, those results corresponding to the baseline mortality of the cold regions population showed by Waldvogel et al. (2018). Apparently, the small number of generations for pre‐exposure did not elicit a benign effect on the fitness trait. Negative pleiotropic interactions to heat resistance or transgenerational epigenetic effect (Heard & Martienssen, 2014; Whittle, Otto, Johnston, & Krochko, 2009) could have caused the heightened mortality at high temperatures. However, since temperature conditions experienced by the next generation are difficult or even impossible to predict for a multivoltine species, such a canalization should be selected against if under genetic control. Nevertheless, it is possible that this difference was induced by the experimental protocol, as the experiment did not include feeding during the 48H of treatment. This could have been detrimental for cold‐raised larvae, because while their developmental rate was increased with high temperature, they lacked the necessary energy. This therefore brings the light on the fact that the higher temperature treatment led to an increase in development speed. This means that larvae subjected to 26°C treatment theoretically developed more during 48 hr than at 14°C and this, without food intake.

The intermediate temperature regime applied for three and five generations did also not result in a relative or absolute decrease in mortality when the offspring was exposed to this temperature and even an increase compared to 14°C regarding the linearity of the results. This is probably because 20°C can be considered as the most benign temperature for the species (Supporting Information Table S1), Therefore it did not apply a large enough selection pressure to overcome the genetic drift in the relatively small experimental populations. The mortality level of 30%–40% observed at this temperature is a known baseline for the species, at least in experimental conditions (Nemec, Heß, Nowak, & Pfenninger, 2012; Nowak, Jost, et al., 2007; Vogt, Nowak, et al., 2007; Vogt, Pupp, et al., 2007; Waldvogel et al., 2018). The slightly, albeit marginally nonsignificant increase in mortality at 26°C is potentially due to a stronger selection pressure at this temperature.

The pre‐exposure to 26°C yielded surprising results, because it reduced mortality significantly and substantially down to about 12% in all treatment temperatures despite the difference in developmental speed between the treatment temperatures (Figure 5 right panel) contradicting the baseline mortality of warm region population from Waldvogel et al. (2018). The fact that there is no variation among the treatments, that is, a lack of interaction between the genotypes with the respective environment argues for a deterministic, that is, genetic effect. It is, however, unclear why mortality is not generally so low in *C. riparius*, if it can demonstrably be swiftly achieved by selection. Trade‐offs with other unmeasured, costly fitness traits appear a reasonable explanation. Another indication for selective processes was the obvious difference in variance within treatments: In PET_14°_ and PET_20°_, the variance of mortality is high, indicating that a lot of variation is present within replicates, while it is strongly reduced for PET_26°_, which can be expected under selection of resilient phenotypes (Bettencourt, Kim, Hoffmann, Feder, & Noor, 2002; Cavicchi, Guerra, La Torre, & Huey, 1995; Debat & Le Rouzic, 2018). We thus hypothesize that this adaptation is based on standing variation given moderate number of individuals, the low mutation rate of our species (Oppold & Pfenninger, 2017), the identical response of all replicates, and the short pre‐exposure time (five generations) which would not be long enough to see de novo mutations rising to appreciable frequencies in the population. Our results are consistent with studies in *Drosophila melanogaster*. Populations reared at 28°C survived better when compared to populations reared at lower temperature (Cavicchi et al., 1995). The same laboratory populations were later found to have fixed alleles of the heat shock Hsp70 gene, but also a lower level of inducible heat shock proteins at 28°C than at lower TTs (Bettencourt et al., 2002). This lower level of inducible heat shock proteins was interpreted as lower capability to respond via phenotypical plasticity to temperature increase (Bettencourt, Feder, & Cavicchi, 1999); following the hypothesis that in this case, *T*_opt_ has shifted to 28°C. A similar pattern was also found in a natural *D*. *melanogaster *strain from Africa displaying an exceptional tolerance toward high temperatures (Zatsepina et al., 2001). If the adaptation mechanisms are the same in *C. riparius*, they can have an important impact on its population dynamics since climatic models have predicted an increase in temperature by at least 2.6°C. An adaptation to high temperature would then increase their metabolism as well as their developmental speed and thus number of generations without increasing mortality. This could lead to “midge blooms” during summer by increasing population size. This in turn could create problems for human health according to previous findings that Chironomids may be allergenic for humans (Eriksson, Ryden, & Jonsson, 1989; Tee et al., 1985; Tee, Cranston, & Kay, 1987) and are able to spread diseases as such as cholera (Broza & Halpern, 2001; Halpern, 2011).

Our results suggest that (a) rapid adaptation to different temperature regimes is possible in *C. riparius *within a few generations and (b) therefore the response to seasonal changes in the temperature regime of natural populations may be at least in part be driven by adaptation and not only by phenotypic plasticity. However, our experiments have been conducted on a mixture of populations from across Europe. This mixture perhaps resulted in an artificial increase in genetic diversity composed of warm and cold temperature adapted individuals. Therefore, even if it was shown that many of these haplotypes may occur together in natural populations because of high gene flow (Waldvogel et al., 2018), the adaptation noticed during our experiment may not be as fast under natural conditions. Still, previous studies indicated that field populations have a high genetic variability (Waldvogel et al., 2018). On the other hand, mixing of different populations may have led to outbreeding depression by induction of intrinsic genetic incompatibilities which, in turn, could have slowed or masked adaptation to the external selection regime (Lynch, 1991). Such outbreeding depression has been observed in crossings of different *C. riparius *populations (Oppold et al., 2017). In our experiment, we only studied mortality as response to temperature; this may have biased our results by overseeing some responses that could have change the overall interpretation of the adaptation mechanisms. Therefore, to strengthen the inference of rapid adaptation over seasonal timescales further, it needs to be shown that similar phenotypic changes also occur in natural populations and that these changes are linked or at least associated with respective genomic allele frequency changes.

## 5 CONCLUSION

In this study, we showed that *C. riparius* lowered mortality due to high‐temperature exposure by rapid adaptation rather than phenotypic plasticity. Our results thus indicate that *C. riparius* is able to react to seasonally varying temperature regimes by rapid adaption within a few generations, modifying the survival of the organism not only for warm but also for colder temperatures. This study adds to the currently accumulating literature of rapid adaptation in multivoltine species.

## CONFLICT OF INTERESTS

The authors declare that they have no competing interests.

## AUTHOR CONTRIBUTION

QF, AW, and MP conceived the project, MP supplied animals, facilities, material, and equipment, QF, AMW, and MP designed the experiments, QF conducted the experiments, analyzed the data, and drafted the manuscript. AW helped conducting the experiments. BF provided feedback on the analysis. All authors contributed to manuscript writing and approved the final version of the manuscript.

## DATA ACCESSIBILITY

The data sets supporting this article are available from the Dryad Digital Repository. https://doi.org/10.5061/dryad.d5b598n.
