# Supplementary material for: Rapid adaptation to high temperatures in Chironomus riparius
Source: Ecol Evol. 2018 Dec 3;8(24):12780–9. doi: 10.1002/ece3.4706 (PMC6308882; doi:10.1002/ece3.4706)

**Figure S1:** Macro-photograph of a male adult *Chironomus riparius* (source: Markus Pfenninger)

**Table S1:** Record for the sampling stations of the maximal, minimal and average temperature (°C) per month for the last fifty years based on the WorldClim v1.4 database

**Figure S2:** Mean larval mortality after 48h depending on the delta temperature (°C) (delta temperature = TT– PET) for the third (gray line) and fifth (black dashed line) generations. Letters denote significant mortality differences between TT for the third (Kruskal-Wallis χ^2^_4_, _85_= 22.149, p-value = 8.347x10^-4^) and the fifth generation (Kruskal-Wallis χ^2^_4_, _85_= 51.274, p-value = 1.957x10^-10^).

**Figure S3:** line plot displaying larval mortality after 48h by TT (°C) for the gerenration 3 (A,B,C) and generation 5 (D,E,F). Letters denote significant mortality differences between TT: (A) PET 14 °C (ANOVA F_2,57_= 9.78, p-value = 6.38x10^-4)^, (B) PET 20 °C (ANOVA F_2,19_= 1.219, p-value = 0.31), (C) PET 26 °C (ANOVA F_2,19_=3.11, p-value = 0.06) ,(D) PET 14 °C (ANOVA F_2,57_= 24.54 , p-value = 8.44x10^-7^) (E) PET 20 °C (ANOVA F_2,19_= 4.028, p-value = 0.029) ,(F) PET 26 °C (ANOVA F_2,19_= 0.128 , p-value = 0.88)

**Figure S1**


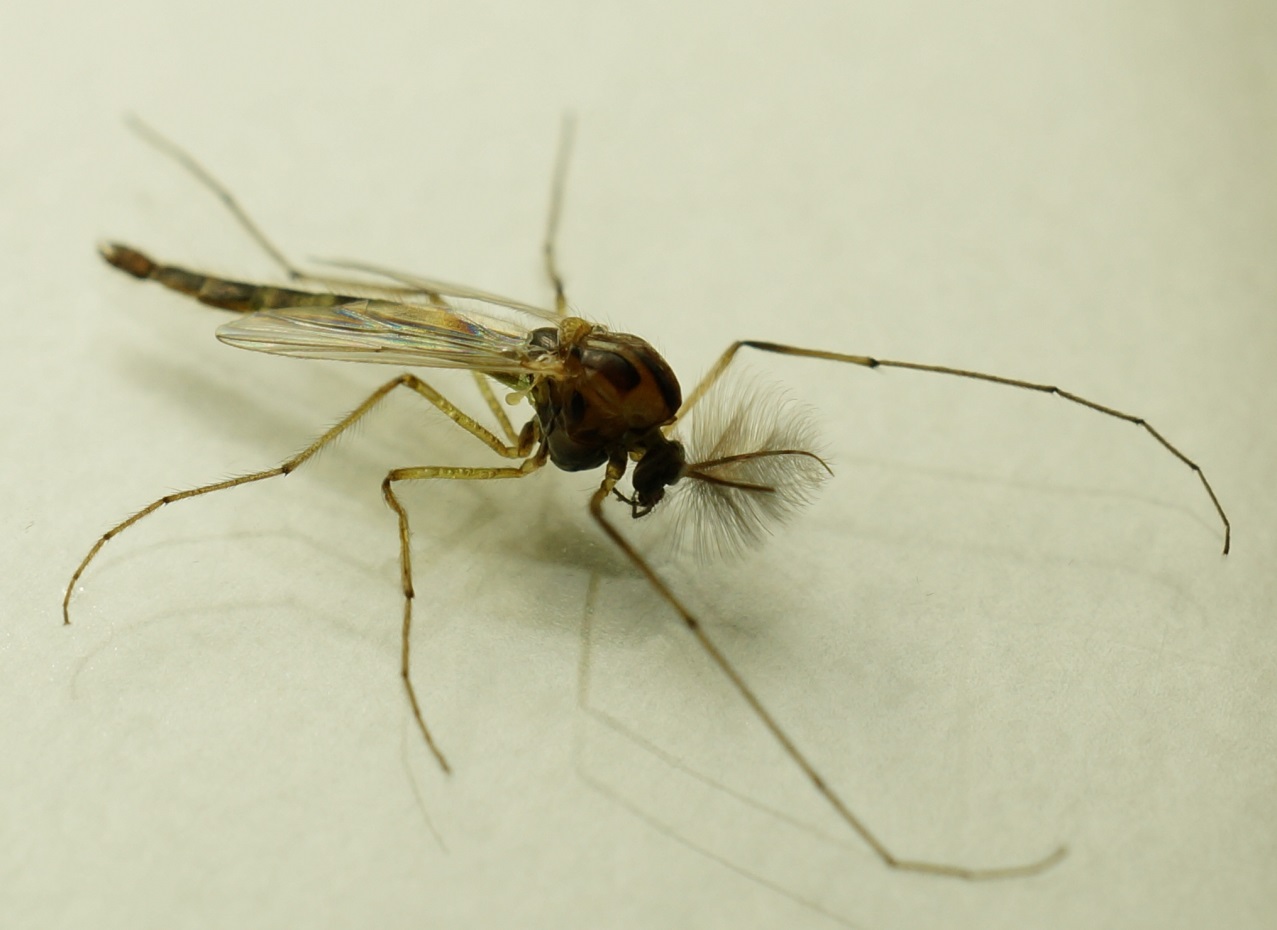


**Table S1**

| MAXIMUM | coordinates | | annual | jan | feb | mar | apr | mai | jun | jul | aug | sep | oct | nov | dec |
| --- | --- | --- | --- | --- | --- | --- | --- | --- | --- | --- | --- | --- | --- | --- | --- |
| SSA | 37.25387 | -4.54135 | 22.19167 | 13.5 | 15 | 17.5 | 20.2 | 23.7 | 28.9 | 32.6 | 32.5 | 28.4 | 22.7 | 17.4 | 13.9 |
| MF | 45.86168 | 4.8865 | 15.625 | 5.1 | 7.1 | 12.2 | 15.6 | 19.8 | 23.4 | 26 | 25.3 | 21.9 | 15.8 | 9.7 | 5.6 |
| NMF | 49.17654 | 6.215667 | 14.15833 | 3.8 | 5.9 | 10.1 | 14.1 | 18.6 | 21.9 | 24 | 23.4 | 20.2 | 14.7 | 8.3 | 4.9 |
| SI | 45.40362 | 8.347332 | 16.85 | 5.3 | 8.1 | 12.7 | 16.9 | 21.2 | 25.2 | 28 | 27 | 23.4 | 17.4 | 10.7 | 6.3 |
| MG | 50.16806 | 9.081927 | 14.15833 | 3 | 4.9 | 10.1 | 15 | 19.4 | 22.8 | 24.3 | 23.5 | 20.3 | 14.2 | 7.9 | 4.5 |
| SSB | 37.39908 | -4.5268 | 22.10833 | 12.8 | 14.6 | 17.2 | 20 | 23.7 | 29.3 | 33.4 | 33 | 28.6 | 22.5 | 16.9 | 13.3 |
|  |  |  |  |  |  |  |  |  |  |  |  |  |  |  |  |
| AVERAGE |  |  |  |  |  |  |  |  |  |  |  |  |  |  |  |
| SSA | 37.25387 | -4.54135 | 16.59 | 8.8 | 10.1 | 12.3 | 14.6 | 17.8 | 22.5 | 25.8 | 25.8 | 22.3 | 17.4 | 12.6 | 9.4 |
| MF | 45.86168 | 4.8865 | 11.01 | 2 | 3.4 | 7.3 | 10.4 | 14.3 | 17.9 | 20.2 | 19.6 | 16.6 | 11.4 | 6.4 | 2.9 |
| NMF | 49.17654 | 6.215667 | 9.87 | 1.4 | 2.7 | 5.8 | 9.2 | 13.3 | 16.6 | 18.4 | 17.9 | 15.1 | 10.5 | 5.4 | 2.6 |
| SI | 45.40362 | 8.347332 | 12.06 | 1.6 | 3.9 | 7.8 | 11.7 | 16 | 19.7 | 22.3 | 21.5 | 18.2 | 12.7 | 6.9 | 2.7 |
| MG | 50.16806 | 9.081927 | 9.74 | 0.3 | 1.5 | 5.6 | 9.7 | 13.8 | 17.2 | 18.8 | 18.1 | 14.9 | 10.1 | 5.1 | 2 |
| SSB | 37.39908 | -4.5268 | 16.47 | 8.4 | 9.8 | 12.1 | 14.5 | 17.8 | 22.6 | 26 | 25.9 | 22.4 | 17.2 | 12.3 | 9 |
|  |  |  |  |  |  |  |  |  |  |  |  |  |  |  |  |
| MINIMUM |  |  |  |  |  |  |  |  |  |  |  |  |  |  |  |
| SSA | 37.25387 | -4.54135 | 11.80833 | 4.9 | 5.7 | 7.8 | 9.6 | 12.4 | 19.3 | 19.1 | 19.3 | 16.8 | 12.7 | 8.4 | 5.7 |
| MF | 45.86168 | 4.8865 | 6.333333 | -1.2 | -0.5 | 2.3 | 5 | 8.8 | 12.2 | 14.3 | 13.8 | 11.3 | 6.9 | 3.1 | 0 |
| NMF | 49.17654 | 6.215667 | 5.666667 | -1 | -0.4 | 1.5 | 4.2 | 7.9 | 11.2 | 13 | 12.6 | 9.9 | 6.3 | 2.5 | 0.3 |
| SI | 45.40362 | 8.347332 | 7.241667 | -2.2 | -0.4 | 2.9 | 6.5 | 10.7 | 14.1 | 16.5 | 15.9 | 12.9 | 7.9 | 3 | -0.9 |
| MG | 50.16806 | 9.081927 | 5.391667 | -2.3 | -1.8 | 1 | 4.6 | 8.2 | 11.6 | 13.4 | 12.6 | 9.7 | 5.9 | 2.2 | -0.4 |
| SSB | 37.39908 | -4.5268 | 11.36667 | 4.3 | 5.2 | 7.3 | 9.1 | 12 | 19 | 19 | 19 | 16.4 | 12.2 | 7.8 | 5.1 |

**Figure S2**


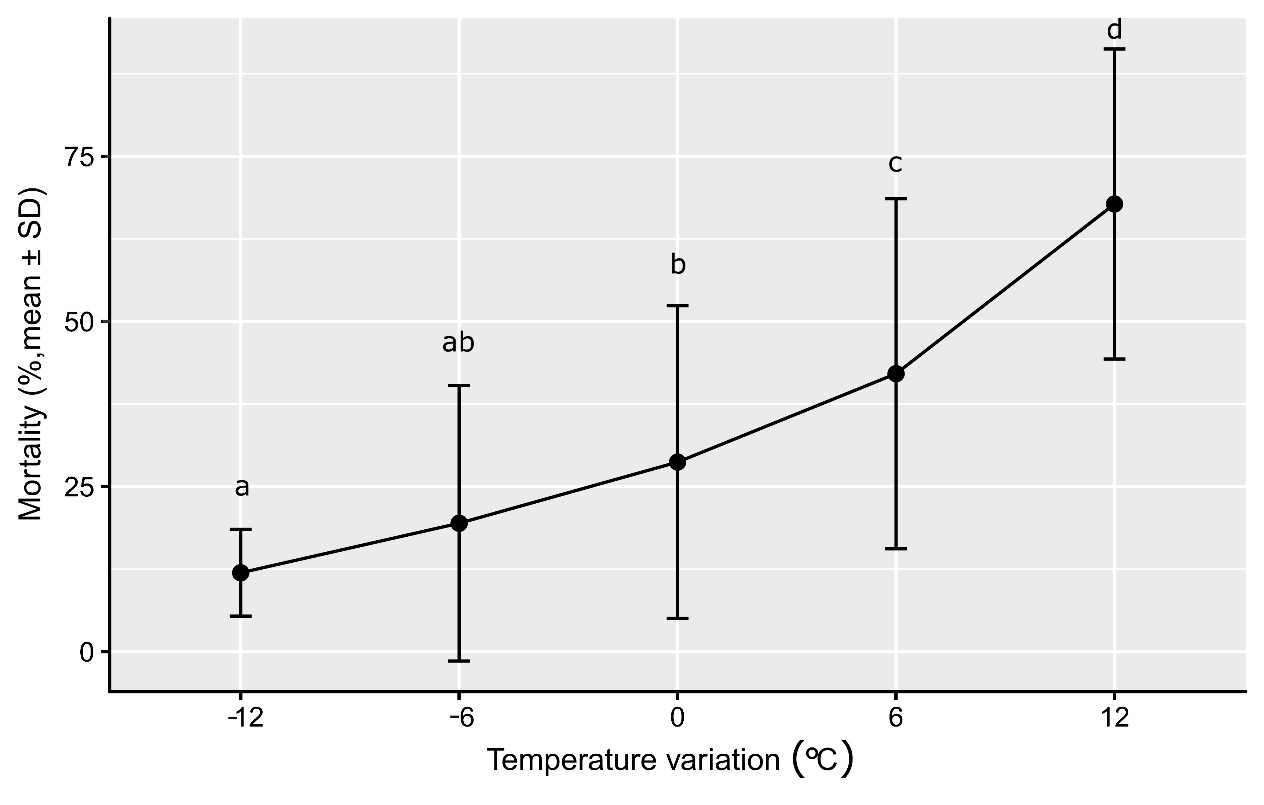

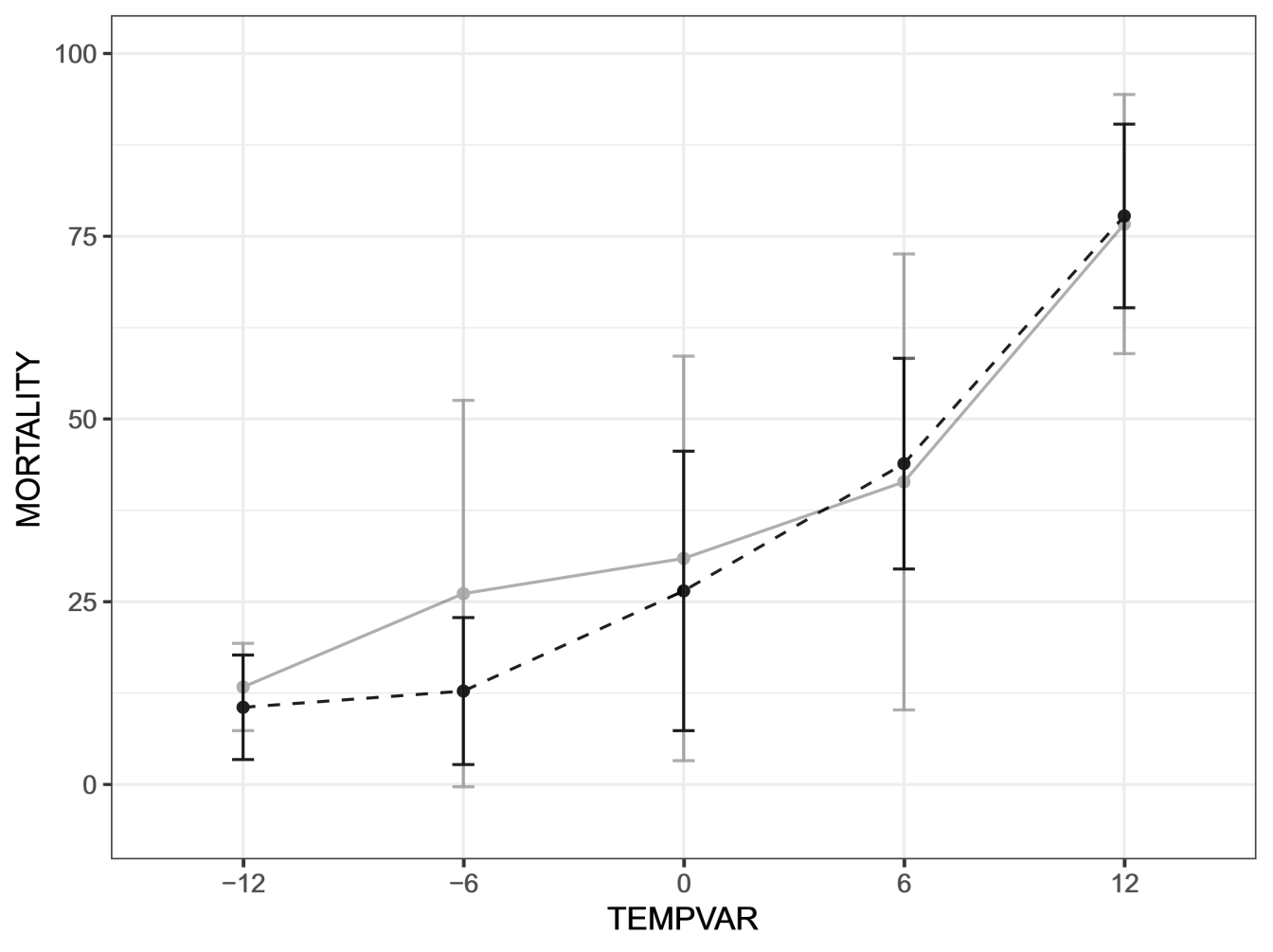

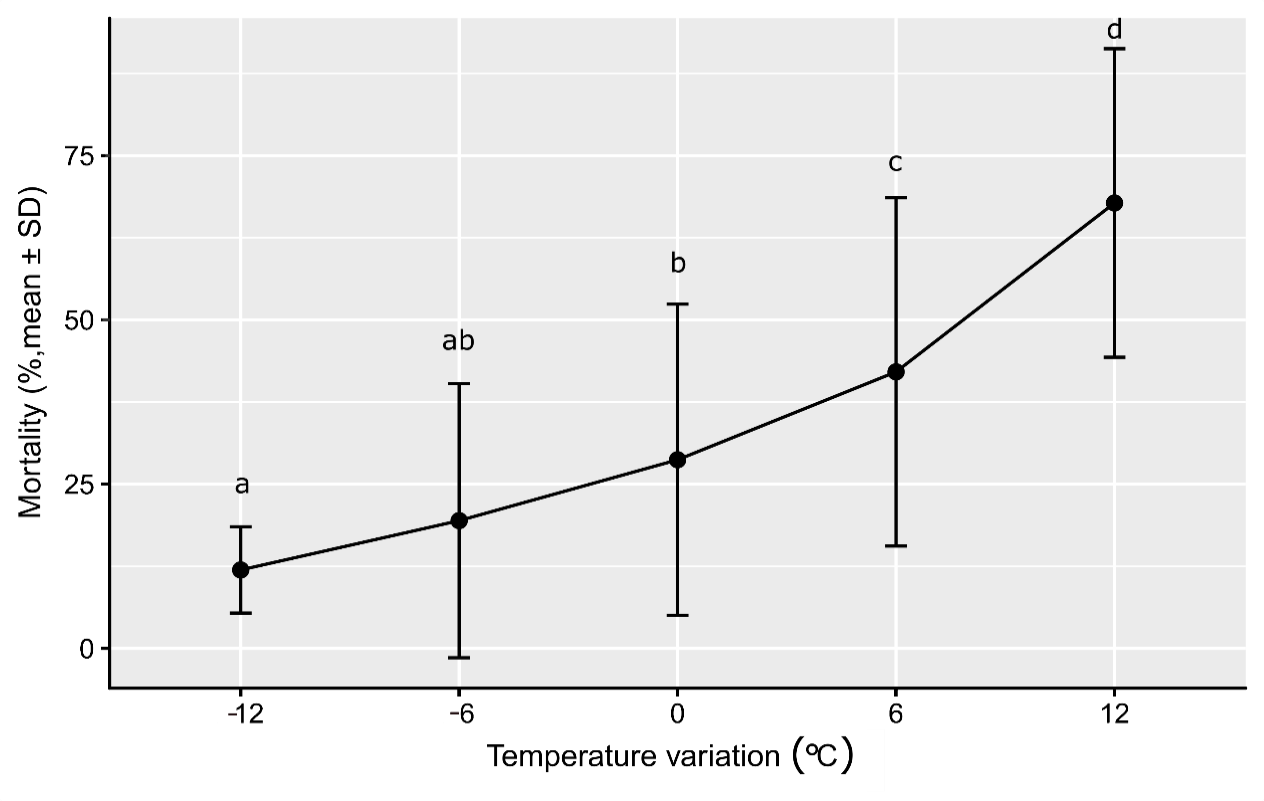


a

a

a

a

a

a

b

ab

c

bc

**Figure S3**


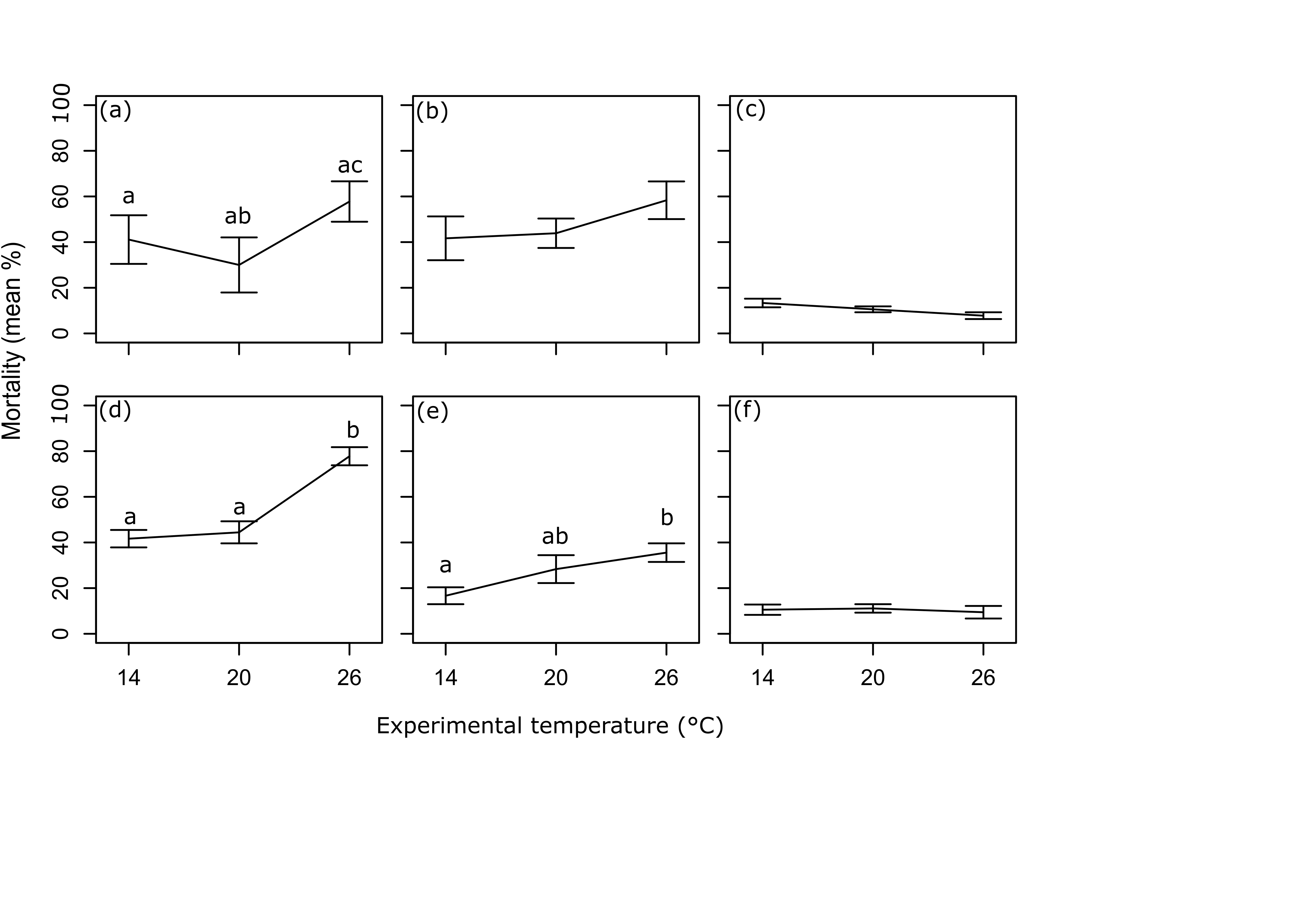

Supplement: Supplementary file 2 [file ECE3-8-12780-s002.docx]
